# Transfused Red Blood Cell Characteristics and Kidney Transplant Outcomes Among Patients Receiving Early Posttransplant Transfusion

**DOI:** 10.1001/jamanetworkopen.2023.32821

**Published:** 2023-09-14

**Authors:** Emilie Gaiffe, Dewi Vernerey, Laurent Bardiaux, Franck Leroux, Aurelia Meurisse, Jamal Bamoulid, Cecile Courivaud, Philippe Saas, Marc Hazzan, Pierre Tiberghien, Didier Ducloux

**Affiliations:** 1 Franche-Comté University, Besançon University Hospital, Etablissement Français du Sang Bourgogne Franche-Comté, INSERM, Unité Mixte de Recherche 1098, RIGHT Interactions Hôte-Greffon-Tumeur/Ingénierie Cellulaire et Génique, Besançon, France; 2 Methodology and Quality of Life in Oncology Unit, Centre Hospitalier Universitaire Besançon, Besançon, France; 3 Etablissement Français du Sang, Toulouse, France; 4 INSERM CIC-1431, Centre Hospitalier Universitaire Besançon, Besançon, France; 5 Service de Néphrologie, Centre Hospitalier Universitaire Besançon, Besançon, France; 6 Etablissement Français du Sang, La Plaine St Denis, France; 7 Nephrology and Transplant department, Lille University Hospital, Lille, France

## Abstract

**Importance:**

Red blood cell transfusion (RBCT) is frequently required in the early post–kidney transplant period, but long-term outcomes associated with RBCT is controversial. Therefore, it may be relevant to investigate the association between RBCT characteristics and transplant outcomes.

**Objective:**

To study the association between RBC storage duration and transplant outcomes.

**Design, Setting, and Participants:**

This was a nationwide retrospective cohort study based on data linking between 2 prospective French nationwide registries. Clinical transplant parameters, outcomes, and RBCT characteristics were extracted from the CRISTAL registry of the Agence de la Biomédecine and the national database of the Etablissement Français du Sang. All 12 559 patients having received a first kidney transplant in France between January 1, 2002, and December 31, 2008, were included. Patients were followed up from transplant to graft loss, death with a functional graft, or data retrieval in June 2016. Data were analyzed from April 2019 to June 2022.

**Exposures:**

Clinical outcomes of transplant recipients who underwent early RBCT were analyzed considering transfusion characteristics.

**Main Outcomes and Measures:**

Cox proportional hazards regression models were fitted to evaluate transplant failure defined as graft loss or death with a functional graft.

**Results:**

Among 12 559 patients who underwent kidney transplant, 3483 received an RBCT during the first 14 days posttransplant. The median (IQR) age of patients was 53.0 (41.5-61.2) years, and 1929 patients (55.4%) were male. Median (IQR) follow-up was 7.8 (7.6-8.0) years. In multivariable analysis, longer (vs shorter) storage duration of transfused RBC was associated with a decrease in risk of transplant failure (hazard ratio, 0.99; 95% CI, 0.98-1.00 for each additional storage day; *P* = .06). Patients transfused with at least 1 RBC unit stored for more than 20 days had a 5% absolute decrease in transplant failure at 3 years and 7% at 5 years compared with those who received RBC stored for less than 20 days.

**Conclusions and Relevance:**

In this study, longer RBC storage duration was associated with a decreased risk of transplant failure among patients who received kidney transplants and RBC transfusions. Preferential use of RBC with longer storage duration might improve kidney graft survival following transplant and transfusion.

## Key Points

**Question:**

Does red blood cell (RBC) storage duration affect transplant failure in patients who received kidney transplants and early posttransplant transfusions?

**Findings:**

In this cohort study including 12 559 kidney transplant recipients, longer RBC storage was associated with longer transplant survival among patients receiving early posttransplant transfusion.

**Meaning:**

Preferential use of the RBC unit with the longest storage duration might reduce the deleterious association between transfusion and kidney graft survival.

## Teaser

This cohort study evaluates associations between duration of red blood cell storage and kidney transplant outcomes among patients receiving early posttransplant transfusion.

## Introduction

Patients who undergo kidney transplant frequently require blood transfusion in the early period after transplantation.^1^ In that event, red blood cell (RBC) units are transfused according to transplant recommendations and current transfusion practices. Several contradictory publications have focused on the impact of posttransplant transfusion, questioning transfusion-mediated development of anti–human leukocyte antigen (HLA) antibodies and antibody-mediated rejection in presensitized patients.^2,3,4,5,6^ Using a national cohort of more than 12 000 patients, we recently reported^7^ that red blood cell transfusion (RBCT) early after transplantation was associated with transplant failure.

Nevertheless, this question may seem trivial. Indeed, when indicated, RBCT is likely to be unavoidable. Consequently, evaluating the characteristics of the RBC units seems to be a more pragmatic strategy to reduce the potential harmful effects of transfusion.

Regardless of the number of RBC units transfused, which also remains a required parameter, characteristics of blood donors, including age, sex, and blood group, have been studied.^8,9,10^ The settings directly related to the method of preparation, storage conservation, or rejuvenation of the RBC units have also been the subject of numerous studies in clinical settings.^11,12^

Duration of storage is one of the most studied and debated product characteristics.^13,14,15^ In most jurisdictions, RBC can be stored up to 42 days before transfusion. Longer storage induces biochemical and morphological modifications of RBC units and accumulation of soluble components that may be deleterious, notably by impairing oxygen delivery to tissues.^13,14,16,17^ Consistent with this, some studies suggested that longer RBC storage was associated with worse outcomes in different clinical settings.^16,18,19^ However, these results have not been consistent, and the benefits of using fresh RBC have been called into question.^20,21,22,23^

Indeed, fresh RBC units contain increased quantity of different mediators, including damaged leukocytes and platelets, cell-free DNA, and microvesicles, that can result in inflammation, coagulation, endothelial injury, and organ damage as well.^11,24^ Moreover, in the specific setting of transplantation, some immunosuppressive effects of RBC may be enhanced by storage.^25,26^ Yet, to our knowledge, no data concerning the putative impact of storage duration of transfused RBC units are available in kidney transplantation.

The principal aim of this study was to investigate the association between duration of RBC storage and transplant outcomes. To perform this study, we linked 2 national databases including all first kidney transplant recipients and all transfusions between 2002 and 2008.

## Methods

### Study Design

We conducted a national longitudinal study to investigate the association between RBC storage duration and transplant outcomes after early posttransplant RBCT in France.^7^ Detailed patient characteristics and blood product characteristics for each unit of transfused blood were extracted from national databases. This study was approved by all French regulatory authorities (Committee for Personal Protection Great East II in Besançon, Advisory Committee on Information Processing for Research in the Field of Health, and French National Computers and Freedom Commission). The exemption of written informed consent was guaranteed by the Advisory Committee on Information Processing for Research in the Field of Health. The study followed the Strengthening the Reporting of Observational Studies in Epidemiology (STROBE) reporting guideline.

### Patients and Blood Products

Transfusion within 14 days posttransplant was completed for each first kidney transplant graft between January 1, 2002, and December 31, 2008. Demographic and clinical data were extracted from the national kidney transplant database CRISTAL. Patients were followed up from inclusion on the transplant waiting list to graft loss, death, or data retrieval in June 2016. Early graft failures, within 15 days posttransplant, were excluded from analysis. All RBC concentrates were produced by the Etablissement Français du Sang and underwent prestorage deleukocytation (<1 × 10^6^ leucocytes/RBC unit). Data collected included blood donor demographic characteristics (including age and sex) as well as blood product characteristics for each RBCT.

A transfusion episode was defined as consecutive transfusions whose interval did not exceed 48 hours. Patients could receive more than 1 RBCT from more than 1 donor. To accommodate this situation, characteristics of RBCT episodes and units, such as the delay from transplant to RBCT, unit storage duration, and RBC donor age, were described by the median, mean, maximum, and minimum values. Examples of each parameter are in the eMethods in Supplement 1.

### Study Outcomes

The primary outcome was the association between RBC storage duration and transplant failure–free survival defined as the time elapsed between transplant and transplant failure. Transplant failure was defined as either graft loss or death with a functioning graft. Graft loss was defined as either return to dialysis or retransplant. The secondary end points were survival without graft loss and survival with a functioning graft.

### Statistical Analysis

Medians with IQRs, means with SEs, and frequencies with percentages were provided for continuous and categorical variables as appropriate. Transplant failure–free survival was estimated using the Kaplan-Meier method and described using median or rate at specific time points with 95% CIs. Follow-up duration was calculated using a reverse Kaplan-Meier estimation.^27^ Restricted cubic spline modeling for continuous parameters was used to investigate their association with transplant failure–free survival and identified cutoff of interest. Cox proportional hazard models were performed to estimate hazard ratios (HRs) and 95% CIs for factors associated with transplant failure.

The association of baseline parameters with transplant failure was first assessed using univariate Cox analyses, and then parameters with *P* values of less than .05 were entered into a final multivariable Cox regression model after considering collinearity among variables with a correlation matrix. A final multivariable Cox regression was proposed. The assumption of proportionality was checked by plotting log minus log survival curves and by cumulative martingale process plots.

Sensitivity analyses with a frailty approach by using a random component for the hazard function based on the transfusion region was applied in the final multivariable Cox model and by considering only the subset of patients having experienced a single transfusion episode of 1 or 2 RBC units.

To model the association of parameters of interest identified in the multivariate model for the primary composite end point (transplant failure–free survival) with each of considered events (graft loss and death during transplant), the Fine and Gray subdistribution hazards approach was applied to address competing risks between graft loss and death during transplant. Cumulative incidence function estimate with subdistribution hazards approach proposed by Fine and Gray was used to model the association of parameters with graft loss–free survival and survival with functional graft.

All analyses were performed using SAS version 9.4 (SAS Institute) and R version 4.1.2 (R Foundation). *P* values less than .05 were considered statistically significant, and all tests were 2-sided. *P* values were not corrected for multiple tests and should be interpreted as exploratory except for the primary objective (the association between RBC storage duration with transplant failure–free survival). Details on the interpretation of important statistical concepts are given in the eMethods in Supplement 1.

## Results

### Population and RBC Units

Our database identified 3483 of 12 559 (28%) patients who received an early posttransplant RBCT. Most had 1 transfusion episode (n = 2512 [72%]), most often consisting of 1 or 2 RBC units (n = 166 [7%] and 1739 [69%], respectively) (eFigure 1 in Supplement 1).

Patient characteristics have been previously described.^10^ Briefly, the median (IQR) age of patients was 53.0 (41.5-61.2) years, and 1929 patients (55.4%) were male. The median (IQR) body mass index was 23.6 (20.9-26.6), calculated as weight in kilograms divided by height in meters squared. Most patients (n = 2997 [86.3%]) were on dialysis before transplant (977 had glomerulopathy [28.1%]), 1433 had CMV [63.8%]), 2192 did not have preimmunization before transplant [87.1%]), and 3310 received an organ from a deceased donor [95.0%]). Median (IQR) follow-up was 7.8 (7.6-8.0) years.

Characteristics of RBC units are summarized in eTable 1 in Supplement 1. The median number of RBC units transfused was 2 (95% CI, 2-4) for a median of 1 episode (95% CI, 1-2) (eTable 1 in Supplement 1). The mean (SD) delay from transplant to issuing of RBC unit for transfusion was 3.5 (4) days. The mean (SD) time for RBCT was 4.6 (3.9) days. Most patients received at least 1 RBC unit from a donor of the opposite sex (n = 2837 [81.5%]), and in half of the cases (n = 1360 [47.9%]) a male recipient received at least 1 transfusion from a female donor (eTable 1 in Supplement 1). As in our previous study,^7^ the frequency of patients receiving at least 1 RBC unit after transplant remained unchanged throughout the study period.

### Association of Duration of RBC Storage and Transplant Failure

In univariate analysis, a longer minimum storage duration of RBC was associated with a reduced risk of transplant failure (hazard ratio [HR], 0.99; 95% CI, 0.98-0.99 for each additional day; *P* < .001). Similar results were obtained using the maximum, median, or mean storage time of RBC transfused per patient and in multivariable analysis with all transfusion characteristics with a *P* value less than .05 in univariate analysis or by selecting the parameters of interest in parsimonious model (Table 1).

**Table 1.** Transfusion Characteristics Associated With Transplant Failure in Patients Who Received Posttransplant Transfusion

Characteristic	Univariate analysis				Full multivariable analysis (n = 3483; 1218 events)^a^		Parsimonious multivariable analysis (n = 3483; 1218 events)	
	No. of patients	No. of events	Hazard ratio (95% CI)	*P* value^b^	Hazard ratio (95% CI)	*P* value^b^	Hazard ratio (95% CI)	*P* value^b^
Transfusion blood group mismatch								
No	3272	1134	1 [Reference]	.55	1 [Reference]	.75	NA	NA
Recipient blood group A	54	21	1.01 (0.66-1.56)		0.96 (0.63-1.48)		NA	NA
Recipient blood group B	80	31	1.25 (0.88-1.77)		1.11 (0.78-1.59)		NA	NA
Recipient blood group AB	77	32	1.16 (0.81-1.65)		1.18 (0.83-1.68)		NA	NA
Blood donor sex mismatch								
No	646	218	1 [Reference]	.20	NA	NA	NA	NA
Yes	2837	1000	1.10 (0.95-1.27)		NA	NA	NA	NA
Blood donor sex mismatch								
No	646	218	1 [Reference]	.06	1 [Reference]	.28	NA	NA
Female blood donor/male recipient	1477	541	1.17 (1.00-1.37)		0.97 (0.82-1.14)		NA	NA
Male blood donor/female recipient	1360	459	1.03 (0.88-1.21)		1.07 (0.91-1.26)		NA	NA
RBC transfusion episodes per patient	3483	1218	1.25 (1.15-1.36)	<.001	1.28 (1.17-1.40)	<.001	1.29 (1.17-1.41)	<.001
RBC transfusion episodes per patient								
1	2512	829	1 [Reference]	<.001	NA	NA	NA	NA
2	757	291	1.28 (1.12-1.46)		NA	NA	NA	NA
>2	214	98	1.59 (1.29-1.96)		NA	NA	NA	NA
RBC transfusion units per patient	3483	1218	1.06 (1.03-1.09)	<.001	NA	NA	NA	NA
RBC transfusion units per patient								
1	166	48	1 [Reference]	.003	NA	NA	NA	NA
2	1749	567	1.11 (0.83-1.50)		NA	NA	NA	NA
3	330	127	1.38 (0.99-1.93)		NA	NA	NA	NA
4	504	197	1.47 (1.07-2.01)		NA	NA	NA	NA
5	144	55	1.43 (0.97-2.11)		NA	NA	NA	NA
6	187	69	1.23 (0.85-1.77)		NA	NA	NA	NA
>6	403	155	1.47 (1.06-2.03)		NA	NA	NA	NA
Delay from transplant to issue of first RBC unit transfusion	3483	1218	1.02 (1.01-1.03)	.004	1.04 (1.02-1.05)	<.001	1.04 (1.02 1.05)	<.001
Delay from transplant to issue of first RBC unit transfusion								
≤0	1163	385	1 [Reference]	.02	NA	NA	NA	NA
>0 ≤ 2	780	282	1.20 (1.03-1.39)		NA	NA	NA	NA
>2 ≤ 6	724	249	1.12 (0.96-1.31)		NA	NA	NA	NA
>6	816	302	1.25 (1.07-1.45)		NA	NA	NA	NA
Delay from transplant to issue of first RBC unit transfusion								
≤0	1163	385	1 [Reference]	.005	NA	NA	NA	NA
>0	2320	833	1.19 (1.05-1.34)		NA	NA	NA	NA
Minimum duration of transfused RBC storage, d	3483	1218	0.99 (0.98-0.99)	<.001	0.99 (0.98-1.00)	.008	1 [Reference]	.006
							0.99 (0.98-1.00)	
Minimum duration of transfused RBC storage, d								
≤9	1014	384	1 [Reference]	.005	NA	NA	NA	NA
>9 ≤ 14	821	295	0.93 (0.80-1.09)		NA	NA	NA	NA
>14 ≤ 20	835	284	0.86 (0.74-1.00)		NA	NA	NA	NA
>20	813	255	0.76 (0.65-0.89)		NA	NA	NA	NA
Minimum duration of transfused red cell storage, d								
≤20	2670	963	1 [Reference]	.003	NA	NA	NA	NA
>20	813	255	0.81 (0.71-0.93)		NA	NA	NA	NA
Minimum blood donor age, y	3483	1218	0.99 (0.99-1.00)	.02	NA	NA	NA	NA
Minimum blood donor age, y								
≤20	1103	409	1 [Reference]	.03	NA	NA	NA	NA
>20- ≤ 25	708	250	0.97 (0.83-1.14)		NA	NA	NA	NA
>25 ≤ 36	815	294	0.95 (0.82-1.10)		NA	NA	NA	NA
>36	857	265	0.80 (0.68-0.93)		NA	NA	NA	NA
Minimum blood donor age, y								
≤36	2626	953	1 [Reference]	.004	0.89 (0.77-1.02)	.09	1 [Reference]	.08
>36	857	265	0.82 (0.71-0.94)				0.88 (0.77-101)	
Mean delay from transplant to issue of RBC unit for transfusion, d	3483	1218	1.16 (1.10-1.22)	<.001	NA	NA	NA	NA
Mean delay from transplant to issue of RBC unit for transfusion, d								
≤1	937	89	1 [Reference]	<.001	NA	NA	NA	NA
>1 ≤ 4	935	304	1.14 (0.97-1.34)		NA	NA	NA	NA
>4 ≤ 7.25	743	288	1.44 (1.22-1.70)		NA	NA	NA	NA
>7.25	868	337	1.46 (1.25-1.71)		NA	NA	NA	NA

Abbreviations: NA, not applicable; RBC, red blood cell.

^a^ The full multivariable Cox model was obtained by entering all parameters, excepting those identified with a strong association (RBC transfusion units were associated with RBC transfusion episodes and mean delay from transplant to issue of RBC unit for transfusion was associated with delay from transplantation to issue of first RBC unit transfusion).

^b^ Cox proportional hazard models were used to estimate associations of parameters with transplant failure–free survival. Values of *P* < .05 were considered statistically significant, and all tests were 2-sided.

Categorization of RBCT characteristics was determined by restricted cubic spline modeling (eFigure 2 in Supplement 1). This model for the minimum duration of RBC storage suggests a cutoff at 20 days for patients with a low risk of transplant failure. This cutoff was confirmed by sensitive methods, including optimal cut-point analysis (cutoff 23 days).^28^ The upper quartile limit for the minimum duration of RBC storage was 20 days.

The blood donor age was also associated with an increased risk of transplant failure. This was especially true with blood donors younger than 36 years (Table 1).

Since transfusion characteristics may greatly vary from one patient to another (eg, in cause, volume, time, and frequency), we studied all available parameters related to transfusion and included them in our model. The number of RBCT episodes and units per patient, the delay between transplant and first RBC unit transfusion, and the mean delay from transplantation to issue of RBC units for transfusion were also associated with an increase in transplant failure (Table 1). In the full multivariable Cox analysis, longer storage duration of transfused RBC was associated with a decrease in risk of transplant failure (hazard ratio, 0.99; 95% CI, 0.98-1.00 for each additional storage day; *P* = .06) A correlation matrix was used to detect significant correlations between investigated parameters to select the most relevant variables (eFigure 3 in Supplement 1).

**Figure 1.**
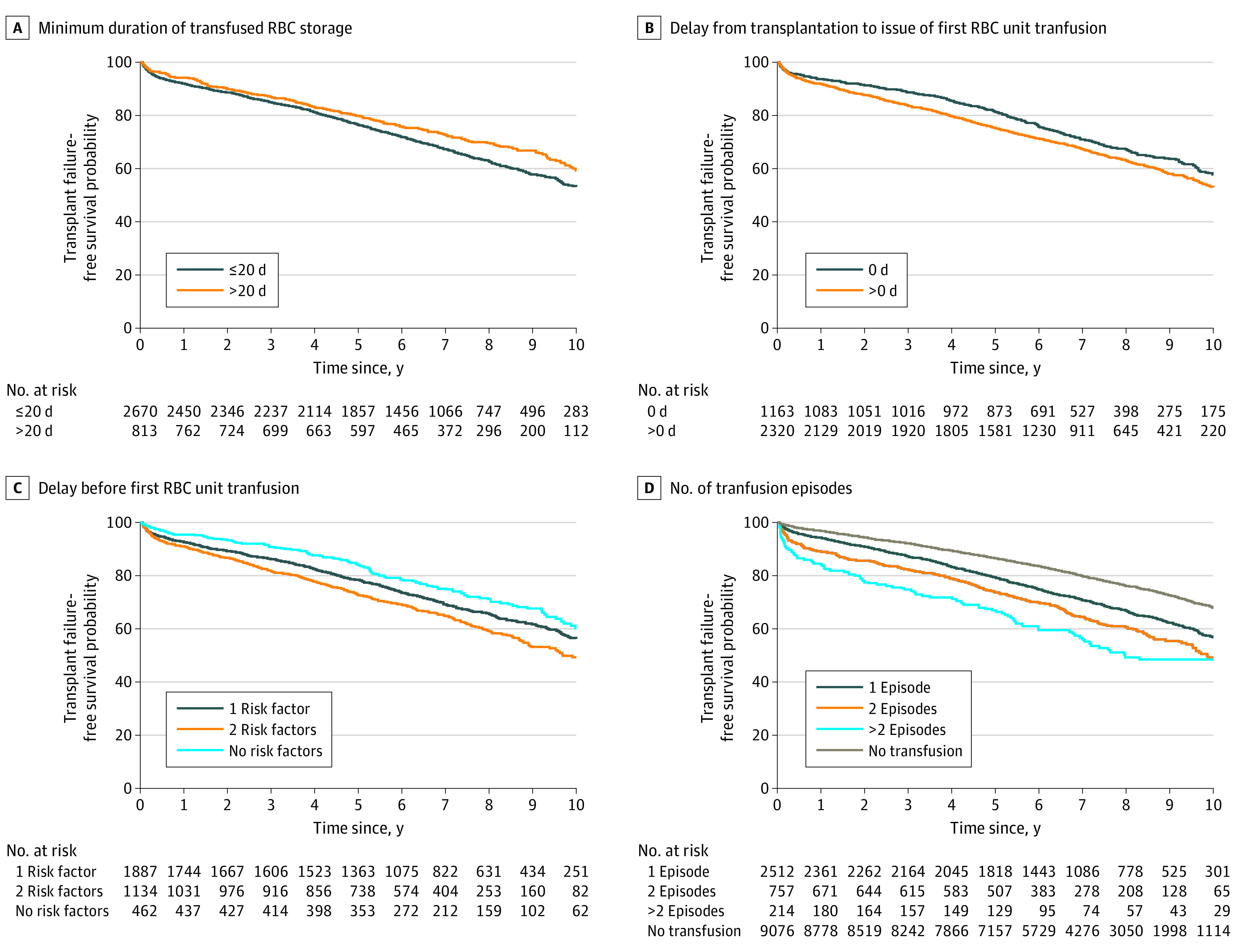
Kaplan-Meier Transplant Failure–Free Survival Curves for Patients According Transfusion Characteristics RBC indicates red blood cell.

The final multivariable Cox analysis included all significant transfusion parameters with the best threshold selected using restricted cubic spleen method and parameters available for most patients that can be associated with graft failure in anterior univariate analyses (Table 2). The use of at least 1 RBC unit with longer duration storage (ie, more than 20 days) was associated with a decrease in risk of transplant failure (HR, 0.84; 95% CI, 0.73-0.96 for each additional day; *P* = .01) (Table 2). Survival analysis showed that, among patients who received transfusions, transfusion with an RBC unit stored for 20 days or more was associated with a 2.1% absolute increase in transplant survival at 1 year and 6% at 5 years (HR, 0.81; 95% CI, 0.79-0.84 and HR, 0.75; 95% CI, 0.73-0.77, respectively; *P* = .003) compared to transfusion with RBC units stored for less than 20 days (Table 2 and Figure 1A).

**Table 2.** Clinical and Transfusion Characteristics of Interest Associated With Transplant Failure in Patients Who Received Posttransplant Transfusion

Multivariable analysis	Final multivariable model (n = 3469; 1208 events)^a^	
	Hazard ratio (95% CI)	*P* value^b^
Recipient age, y	1.02 (1.02-1.03)	<.001
Recipient sex		
Male	1 [Reference]	.52
Female	0.96 (0.86-1.08)	
Dialysis antecedent		
No	1 [Reference]	<.001
Yes	2.03 (1.63-2.52)	
Overall HLA mismatch		
A/B/DR	1.03 (0.99-1.08)	.19
Donor type		
Living	1 [Reference]	.56
Cerebrovascular death	1.21 (0.85-1.72)	
Other cause of death	1.17 (0.49-2.79)	
RBC transfusion episodes per patient		
1	1 [Reference]	<.001
2	1.22 (1.07-1.41)	
>2	1.70 (1.37-2.12)	
Delay from transplant to issue of first RBC unit transfusion		
≤0	1 [Reference]	.002
>0	1.22 (1.07-1.38)	
Minimum duration of transfused RBC storage, d		
≤20	1 [Reference]	.01
>20	0.84 (0.73-0.96)	
Minimum blood donor age, y		
≤36	1 [Reference]	.02
>36	0.85 (0.74-0.98)	

Abbreviation: RBC, red blood cell.

^a^ The final multivariable analysis was obtained by entering all transfusion parameters of the parsimonious multivariable analysis with the best threshold selected using restricted cubic spleen and anterior univariate analyses and all the clinical parameters available for most patients that can influence graft failure.

^b^ Cox proportional hazard models were used to estimate associations of the parameters with transplant failure–free survival. Values of *P* < .05 were considered statistically significant, and all tests were 2-sided.

### Sensitivity Analyses for Association of RBC Storage With Transplant Failure

**Figure 2.**
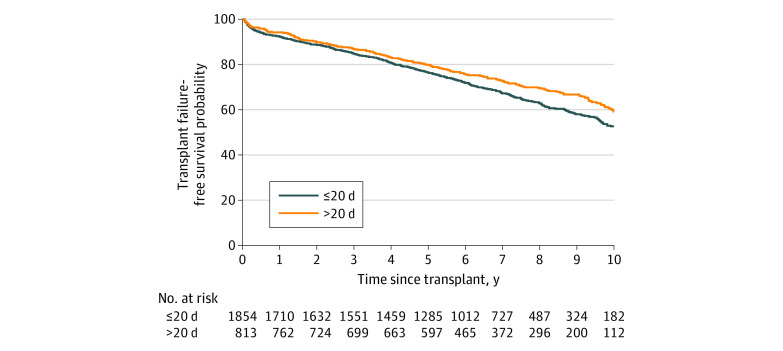
Kaplan-Meier Transplant Survival Curve for Patients According to Red Blood Cell (RBC) Storage Duration Patients who received transfusions with RBC stored for more than 20 days or for 20 days or less were followed up for 10 years.

Results were similar with frailty model analysis based on geographic region (eTable 2 in Supplement 1). These results were also confirmed when comparing patients who received exclusively RBC with storage duration of 20 days or longer to those who received exclusively RBC units with storage duration of less than 20 days (Figure 2).

The delay between transplant and first transfusion remained significantly associated with graft failure in the multivariable analysis (Tables 1 and 2). Moreover, patients who received transfusions the same day as the transplant procedure had a 1.7% absolute decrease in transplant survival at 1 year and 7% at 5 years (HR, 1.19; 95% CI, 1.05-1.34) compared to those who received transfusions later (Figure 1B). We therefore carried out a simultaneous analysis of these 2 risk factors. The delay between transplant and the first RBC unit transfusion and duration of transfused red cells storage revealed that patients who received transfusions on the day of transplant with RBC stored for 20 days or more had 85% higher transplant survival at 5 years than other patients (HR, 1.23; 95% CI, 1.02-1.48) for 1 risk factor vs HR, 1.50; 95% CI, 1.24-1.82 for 2 risk factors; *P* < .001) (Figure 1C).

Furthermore, the number of transfusion episodes was inversely associated with transplant survival (Tables 1 and 2 and Figure 1D). Kaplan-Meier transplant survival curves for patients according to the delay of first RBCT and duration of RBC storage, with 1 transfusion episode or more showed that the number of episodes influenced the associations of the transfusion parameters (eFigure 4 in Supplement 1). To reduce transfusions heterogeneity, we performed the same analyses in a subset of 1905 patients who experienced a single transfusion episode of 1 or 2 RBC units (eTable 3 and eFigure 1 in Supplement 1).

In univariate analysis, the delay between transplant and first RBC unit transfusion, the mean delay from transplant to issue of RBC units for transfusion, and shorter storage duration were also associated with risk of graft failure. As for the overall population, we used a correlation matrix to select the most relevant variable for final multivariable analysis (eTable 3 and eFigure 5 in Supplement 1). The time of first RBC unit transfusion after transplant and shorter storage duration remained significantly associated with transplant failure (eTable 3 in Supplement 1).

### Association of RBCT Characteristics With Graft Loss and Patient Death During Transplant With Competitive Risk Analysis

**Figure 3.**
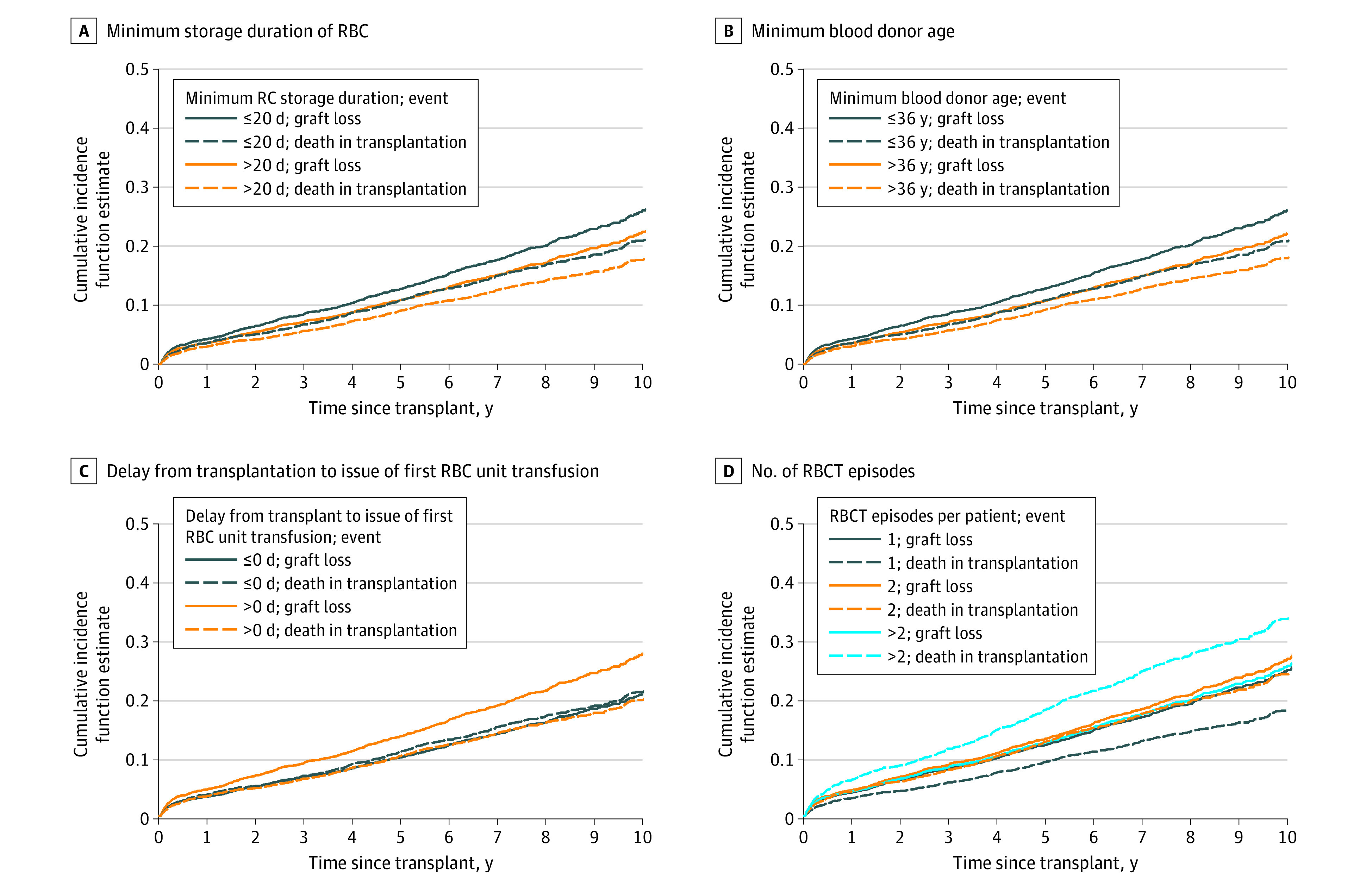
Cumulative Incidence Function Estimate With Fine and Gray Model for Competitive Risk Analysis Model includes 3483 patients, 670 graft loss events, and 548 deaths during transplant. RBC indicates red blood cell; RBCT, red blood cell transfusion.

The associations of RBCT characteristics with graft loss or death during transplant were investigated separately. Similar associations between the minimum storage duration of RBC and the minimum blood donor age were obtained for both outcomes (graft loss and death during transplant) in competitive risk analyses (Figure 3A and B), whereas different outcomes were observed for delay from transplantation and RBCT episodes. Transfusion of the first RBC unit on the day of transplantation (vs later) was associated with better graft survival, and the number of RBCT episodes was associated with death during transplant (Figure 3C and D).

## Discussion

In this cohort study, we report that among patients with kidney transplant and RBC transfusion, those who received RBC with longer storage had better transplant survival. Associations between RBC storage and patient outcomes after transfusion have been long debated. Contrary to our findings, existing literature mainly focuses on deleterious associations between conservation and different end points.^13^ Randomized clinical trials published since 2013 revealed similar conclusions.^18,19,21,22,23^ Nevertheless, the Age of Blood Evaluation (ABLE) study^20^ suggested a trend toward an increased mortality with fresh RBC units. To our knowledge, no data have been reported in kidney transplantation. In liver transplantation, the association of RBC preservation on outcomes is variable but the context is obviously different.^29,30,31^ The conflicting results may suggest that the differential association of RBC storage duration on outcomes could depend on clinical context. In our study, a storage time longer than 20 days was associated with a reduced risk of transplant failure at 5 years by 6% compared to a newer RBC unit (HR, 0.75; 95% CI, 0.73-0.77 vs HR, 0.81; 95% CI, 0.79-0.84). This association was robust and persisted in all sensitivity analyses. Moreover, the size of the association is large, since such a transfusion policy may spare 1 graft loss in the first 5 years posttransplant for 14 patients receiving transfusions during the first 2 weeks after transplant.

The mechanisms underlying the association of RBC storage on graft survival remain speculative. Longer stored RBC may have immunomodulatory properties that would favorably influence graft survival. Different mechanisms are at the origin of this phenomenon, called transfusion-related immunomodulation.^3,32^ Macrophage phenotype switching, reduced expression of class II major histocompatibility complex molecules, and Treg cell activation are some of them.^24,25,33^ The degree of immunosuppression is related to the concentration of soluble HLA class I molecules and Fas ligand in the supernatant, which are both increased by storage duration.^34^ Supernatants of whole RBC inhibit ex vivo phytohemagglutinin-induced tumor necrosis factor–α and interleukin-2 secretion proportionally to the storage time.^35^ Moreover, concentration of many proinflammatory cytokines decreases when storage duration increases.^26^ Accumulation of apoptotic white blood cells during RBC storage may also contribute to immunomodulation.^36^ Some clinical outcomes of transfusion-induced immunosuppression have been previously described. Indeed some studies suggest that RBCT favor recurrence of cancers.^37,38^ Moreover, several studies showed that longer RBC storage is associated with an increased rate of infection.^26,39,40^ Together, these data suggest that longer stored RBC may induce sustained immunomodulation that can favor graft survival.

Alternatively, longer stored RBC may be less likely to cause HLA immunization. RBC units still contain white blood cells even after deleukocytation. White blood cells disappear from RBC units in the first days of storage. However, fresh RBC units containing a greater amount of white blood cells are likely to further expose patients to HLA immunization and subsequent humoral rejection.^2,3,4^

Transfusions on the day of transplant seem to be less deleterious than those performed after. This may be explained by different causes for transfusion in the 2 posttransplant periods. Transfusions on the day of transplant are likely to concern acute bleeding and to be based on clinical appreciation rather than on hemoglobin levels. Those occurring on a later date may be more often related to clinical complications.

Some studies suggest that transfusion sex mismatch might carry a harmful effect.^9,10,41^ Indeed, RBCT from male donors to female recipients may induce immunization against minor histocompatibility antigen H-Y and drive subsequent alloimmune response.^42^ Nevertheless, few data support the clinical relevance of this hypothesis.^15^ In the present study, no association was found between blood transfusion sex mismatch and transplant failure.

### Limitations

This study has limitations. The distribution strategy of RBC favors the oldest unit. In this way, multiple transfusions may increase the likelihood of receiving a fresher RBC unit. Nevertheless, the association of storage duration was adjusted for the number of RBC units transfused as well as the number of transfusion episodes. Moreover, the same association of storage duration was observed in patients who had a single episode of RBCT. Although most transfusions (72%) concerned 1 or 2 RBC units, we and others reported that the harmful association of the transfusion was proportional to the number RBC unit transfused.^7,43,44,45,46^ The association between the need for a massive transfusion and severe posttransplant complications is obvious. However, the deleterious association of the transfusion starts with the first transfusion episode (1 or 2 RBC units). In previous studies, this association only appeared after the third RBC unit.^7,45^ The difference may either be related to a smaller number of patients or different transfusion practices in these studies. The association of time elapsed between transplant and transfusion is also probably influenced by differences in the context and indication of transfusion.

In addition, we have not taken into account an exhaustive list of all the parameters related to blood donors, such as race and ethnicity, previous pregnancies, or genetic characteristics (ie, glucose-6-phosphate dehydrogenase deficiency). These parameters may influence the probability of alloantigen formation as well as the quality of preserved blood, impacting the state of conservation of RBCs.^15,35,36,37,38^ Likewise, other unmeasured parameters, such as organ donor age, delayed graft function, or the presence of donor-specific antibodies, may have an impact on graft failure. However, these factors do not explain the differences observed in this population.

The method of whole blood processing (red-cell filtered RBCs or whole-blood filtered RBCs) is variable from one blood transfusion center to another. Some studies suggest that RBC processing methods may influence RBC quality and patient outcomes.^19^ Nevertheless, we did not observe any differences in the rate of transfusion or in the quality of delivered blood products between regions.

## Conclusions

The findings in this study show an association between longer RBC storage duration and improved graft survival. These results may support the use of the oldest RBC available for transfusion following kidney transplants.

## Notes

Supplement 1.
eMethods.
**eTable 1.** Transfusion characteristics in patients transfused early after transplantation **eTable 2.** Multivariate analysis of transfusion parameters associated with transplant failure with a frailty approach by using a random component for the hazard function based on the transfusion region **eTable 3.** Transfusion characteristics associated with transplant failure for transfused patients with a single transfusion episode (one or two RBC units) **eFigure 1.** Study patient flow chart **eFigure 2.** Restricted cubic spline modelization of red-cell transfusion characteristics **eFigure 3.** Correlation matrix between transfusion characteristics associated with transplant failure endpoint in univariate analyses **eFigure 4.** Kaplan-Meier transplant survival curves for patients according delay from Tx to issue of first red-cell unit transfusion or/and minimum duration of transfused red cells storage for transfused patients with one transfusion episode (A) or more (B) **eFigure 5.** Correlation matrix between transfusion characteristics associated with transplant failure endpoint in univariate for patients who have received one transfusion episode of one or two units

Supplement 2.
Data Sharing Statement
