# Error in Results

**DOI:** 10.1001/jamanetworkopen.2023.41773

**Published:** 2023-10-20

The Original Investigation titled “Transfused Red Blood Cell Characteristics and Kidney Transplant Outcomes Among Patients Receiving Early Posttransplant Transfusion,”^1^ published on September 14, 2023, was corrected to fix typographical errors in the sensitivity analyses in the Results. The article reported that “patients who received transfusions the same day as the transplant procedure had a 1.7% absolute decrease in transplant survival at 1 year.” This has been corrected to “1.7% absolute increase.” It was also reported that “patients who received transfusions on the day of transplant with RBC stored for 20 days or more had 85% higher transplant survival at 5 years.” The correct percentage is 8.5%.
