# Supplementary material for: Transfused Red Blood Cell Characteristics and Kidney Transplant Outcomes Among Patients Receiving Early Posttransplant Transfusion
Source: JAMA Netw Open. 2023 Sep 14;6(9):e2332821. doi: 10.1001/jamanetworkopen.2023.32821 (PMC10502525; doi:10.1001/jamanetworkopen.2023.32821)
Supplement: Supplement 1. — eMethods. eTable 1. Transfusion characteristics in patients transfused early after transplantation eTable 2. Multivariate analysis of transfusion parameters associated with transplant failure with a frailty approach by using a random component for the hazard function based on the transfusion region eTable 3. Transfusion characteristics associated with transplant failure for transfused patients with a single transfusion episode (one or two RBC units) eFigure 1. Study patient flow chart eFigure 2. Restricted cubic spline modelization of red-cell transfusion characteristics eFigure 3. Correlation matrix between transfusion characteristics associated with transplant failure endpoint in univariate analyses eFigure 4. Kaplan-Meier transplant survival curves for patients according delay from Tx to issue of first red-cell unit transfusion or/and minimum duration of transfused red cells storage for transfused patients with one transfusion episode (A) or more (B) eFigure 5. Correlation matrix between transfusion characteristics associated with transplant failure endpoint in univariate for patients who have received one transfusion episode of one or two units [file jamanetwopen-e2332821-s001.pdf]

Supplemental Online Content

Gaiffe E, Vernerey D, Bardiaux L, et al. Transfused red blood cell characteristics and kidney transplant outcomes among patients receiving early posttransplant transfusion. *JAMA Netw Open*. 2023;6(9):e2332821. doi:10.1001/jamanetworkopen.2023.32821

eMethods

eTable 1. Transfusion characteristics in patients transfused early after transplantation

eTable 2. Multivariate analysis of transfusion parameters associated with transplant failure with a frailty approach by using a random component for the hazard function based on the transfusion region

eTable 3. Transfusion characteristics associated with transplant failure for transfused patients with a single transfusion episode (one or two RBC units).

eFigure 1. Study patient flow chart

eFigure 2. Restricted cubic spline modelization of red-cell transfusion characteristics

eFigure 3. Correlation matrix between transfusion characteristics associated with transplant failure endpoint in univariate analyses

eFigure 4. Kaplan-Meier transplant survival curves for patients according delay from Tx to issue of first red-cell unit transfusion or/and minimum duration of transfused red cells storage for transfused patients with one transfusion episode (A) or more (B).

eFigure 5. Correlation matrix between transfusion characteristics associated with transplant failure endpoint in univariate for patients who have received one transfusion episode of one or two units

This supplemental material has been provided by the authors to give readers additional information about their work.

© 2023 Gaiffe E et al. *JAMA Network Open.*

eMethods

Characteristics of RBC transfusion

Characteristics of RBC transfusion episodes and units, such as the delay from transplant to RBC transfusion, unit storage duration, and RBC donor age and gender, were described by the median, mean, max, and min values, respectively . For example, min/max duration of RBC storage per patient is defined as the RBC unit transfused to a patient with the minimum/maximum duration of storage. Median/Mean duration of RBC storage per patient is defined as the median/mean duration of storage of all RBC units transfused to a patient. Min/Max blood donor age is defined as the younger/older blood donor of all RBC units transfused to a patient. Median/mean blood donor age is defined as the median/mean of blood donor age of all RBC units transfused to a patient. Sex mismatched is defined as the transfusion of at least one RBC unit from a blood transfusion donor with a gender different from the blood transfusion recipient. Blood group mismatched is defined as the transfusion of at least one RBC unit from a blood transfusion donor with a blood group different from the blood transfusion recipient.

© 2023 Gaiffe E et al. *JAMA Network Open.*

eTables

eTable 1. Transfusion characteristics in patients transfused early after transplantation

**Characteristics***	**Patients with transfusion after Tx (n= 3483)**
RBC units transfused per patient,	
mean ±SD	3.8 (3.9)
median (95%CI)	2 (2-4)
RBC transfusion episodes per patient,	
mean ±SD	1.3 (0.6)
median (95%CI)	1.0 (1.0-2.0)
Delay from transplantation to issue of first RBC unit for transfusion, days	
mean ±SD	3.5 (4.0)
median (95%CI)	2 (0-6)
Mean delay from transplantation to issue of RBC unit for transfusion, days	
mean ±SD	4.6 (3.9)
median (95%CI)	4.0 (1.0-7.3)
Median delay from Tx to issue of red-cell unit for transfusion, days	
mean ±SD	*4.5 (4.1)*
median (95%CI)	*3.5 (1.0-7.5)*
Min delay from Tx to issue of red-cell unit for transfusion, days	
mean ±SD	*3.5 (4.0)*
median (95%CI)	*2.0 (0 – 6.0)*
Max delay from Tx to issue of red-cell unit for transfusion, days	
mean ±SD	*5.9 (4.6)*
median (95%CI)	*5.0 (1.0-10.0)*
Min duration of storage of transfused RBCs per patient, days	
mean ±SD	15.1 (8.0)
median (95%CI)	14.0 (9.0-20.0)
Mean duration of storage of transfused red cells per patient, days	
mean ±SD	*17.7 (7.6)*
median (95%CI)	*17.0 (12.0-23.0)*
Median duration of storage of transfused red cells per patient, days	
mean ±SD	*17.5 (7.8)*
median (95%CI)	*16.5 (11.5-23.0)*
Max duration of storage of transfused red cells per patient, days	
mean ±SD	*20.6 (8.8)*
median (95%CI)	*20.0 (14.0- 27.0)*
Min Blood donor age, years	
mean ±SD	28.7 (10.6)
median (95%CI)	25.0 (18.0- 62.0)

© 2023 Gaiffe E et al. *JAMA Network Open.*

Mean blood donor age, years	
mean ±SD	*38.7 (8.9)*
median (95%CI)	*39.0 (18.0-62.5)*
Median blood donor age, years	
mean ±SD	*38.7 (9.7)*
median (95%CI)	*39.5 (18.0-62.5)*
Max blood donor age, years	
mean ±SD	*48.9 (11.2)*
median (95%CI)	*51.0 (18.0- 62.0)*
Sex mismatched, n (%)	2837 (81.5%)
Male recipient / female Blood donor	1360 (47.9%)
Female recipient / male Blood donor	1477 (52.1%)

All multiple parameters were studied using the minimum, maximum, median, and mean values. The most relevant parameters have been retained in first in the table. The other parameters are available in italics. *Missing = 0; Values of P<0.05 were considered statistically significant and all tests were two-sided. Abbreviations: CI=Confidence Interval, IQR=Interquartile Range, RBC=Red Blood Cell, SD=Standard Deviation, Tx=Transplantation.

© 2023 Gaiffe E et al. *JAMA Network Open.*

eTable 2. Multivariate analysis of transfusion parameters associated with transplant failure with a frailty approach by using a random component for the hazard function based on the transfusion region

	Multivariate analysis (N= 3483; 1218 events)	
	**HR (95%CI)**	**p value****
**Red-cell transfusion episodes per patient**	1.317 (1.206; 1.439)	< 0.001
**Delay from Tx to issue of first red-cell unit transfusion**		
<=0	1	
>0	1.336 (1.176; 1.519)	< 0.001
**Min duration of transfused red cells storage, days**		
<=20	1	
>20	0.844 (0.733; 0.971)	0.02

All parameters with p value <0.05 have been retained in models. The number of observations read is 3483 and 1218 events. **Cox-proportional-hazard models used to estimate association of the parameters with TXFFS. Values of P<0.05 were considered statistically significant and all tests were two-sided. CI=Confidence Interval, HR=Hazard Ratio, Min=Minimum, Tx=Transplantation, TXFFS=Transplantation Failure Free Survival.

eTable 3. Transfusion characteristics associated with transplant failure for transfused patients with a single transfusion episode (one or two RBC units)

			Univariate analysis		Full multivariable analysis (N= 1905; 612 events)		Final multivariable analysis (N= 1905; 612 events)	
	**N Patients**	**N event**	**HR (95% CI)**	**p value****	**HR (95% CI)**	**p value****	**HR (95% CI)**	**p value****
**Red-cell units transfused per patient***								
1	166	48	1					
2	1739	564	1.114 (0.830; 1.496)	0.47				
**Delay from Tx to issue of first red-cell unit transfusion***	1905	612	1.024 (1.006; 1.043)	0.01	1.024[1.005; 1.042)	0.01	1.024 (1.006; 1.043)	0.009
**Delay from Tx to issue of first red-cell unit transfusion***								
<=0 <=2	421	134	1					
>0 <=4 >2 <=4	638	193	1.060 (0.851; 1.322)					
>4 >7	846	285	1.223 (0.995; 1.502)	0.11				
**Min duration of transfused red cells storage, days***	1905	612	0.990 (0.981 ; 1.000)	0.05	0.990 (0.981; 1.000)	0.05	0.990 (0.981; 1.000)	0.05
**Max duration of transfused red cells storage, days***	1905	612	0.990 (0.981 ; 0.999)	0.04				
**Mean duration of transfused red cells storage, days***	1905	612	0.990 (0.980 ; 0.999)	0.04				
**Min blood donor age, years***	1905	612	0.996 (0.989 ; 1.003)	0.29				
**Max blood donor age, years***	1905	612	0.997 (0.990 ; 1.003)	0.34				
**Mean blood donor age, years***	1905	612	0.995 (0.988 ; 1.003)	0.25				
**Mean delay from Tx to issue of red-cell unit for transfusion, days ***	1905	612	1.025 (1.006 ; 1.043)	0.008	0.997 (0.990; 1.004)	0.42		
**Min delay from Tx to issue of red-cell unit for transfusion, days ***	1905	612	1.024 (1.006; 1.043)	0.009				
**Max delay from Tx to issue of red-cell unit for transfusion, days ***	1905	612	1.025 (1.006 ; 1.043)	0.008				

The full multivariable Cox model was obtained by entering all parameters with p value <0.05 in the full population model (Table IA). The final multivariable Cox model was obtained by entering all parameters with p value <0.05 in univariate analysis, excepting the parameters identified with a strong correlation (mean delay from transplantation to issue of red-cell unit for transfusion, days). *N missing=0. **Cox-proportional-hazard models used to estimate association of the parameters with transplantation success survival. Values of P<0.05 were considered statistically significant and all tests were two-sided. CI=Confidence Interval, Min=Minimum, HR=Hazard Ratio, Tx=Transplantation.

© 2023 Gaiffe E et al. *JAMA Network Open.*

Supplementary Figures

eFigure 1. Study patient flow chart. Kidney transplant patient received one or more transfusion episode defined as consecutive transfusions whose interval does not exceed 48 hours. Patients for whom follow-up does not reach 14 days post transplantation (post transplantation transfusion study period) are excluded from the study. The figure also indicates whether patients received one or more red blood cell unit and RBCT characteristics.

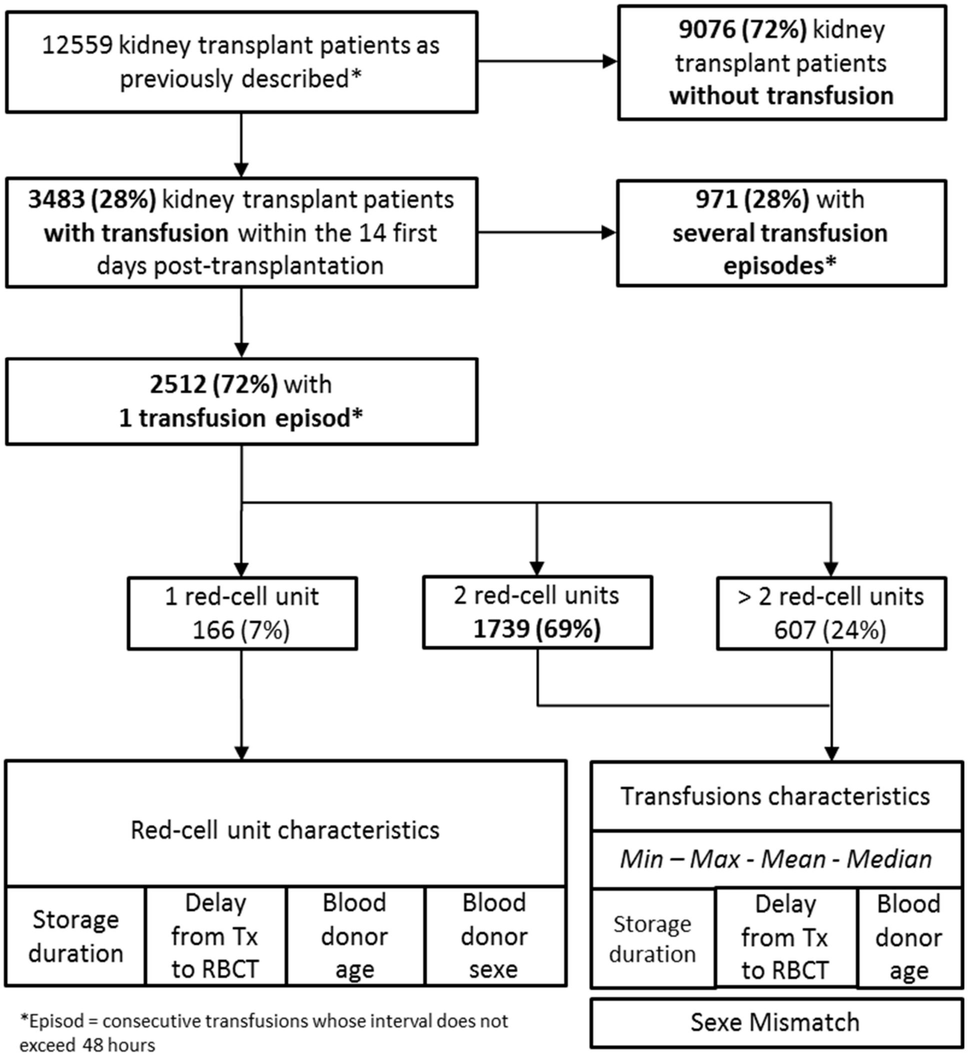

© 2023 Gaiffe E et al. *JAMA Network Open.*

eFigure 2. Restricted cubic spline modelization of red-cell transfusion characteristics: episodes per patient (A), red-cell transfusion units per patient (B), delay from Tx to issue of the first red-cell unit transfusion (C), minimum of duration of transfused red cells storage (D), minimum blood donor age (E), and mean delay from Tx to issue of red-cell unit for transfusion (F) parameters with transplant failure.

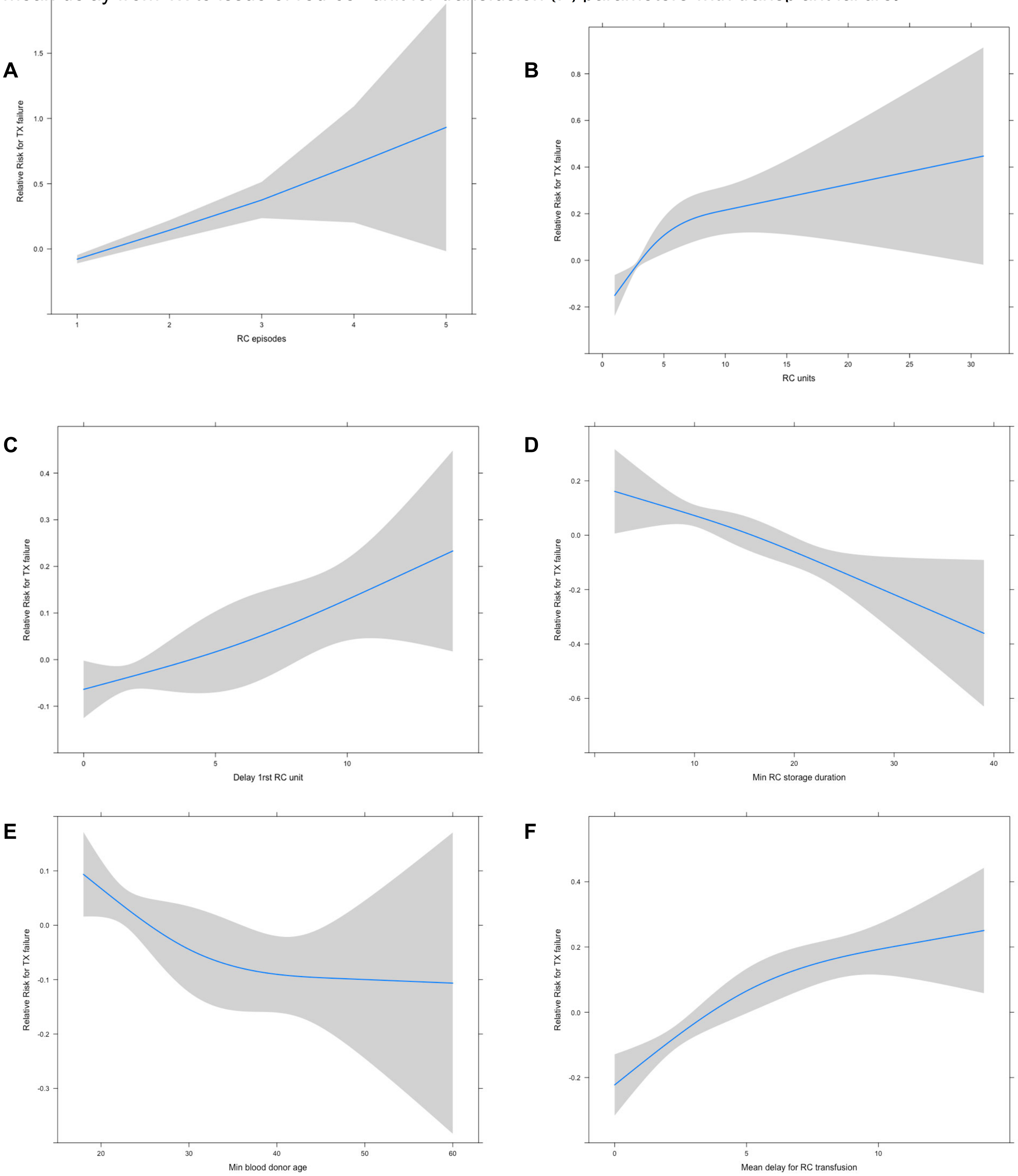

All continuous variables were considered without any transformation. Parameters were analyzed continuously, by median, and quartile for modeling purposes as shown in table 1. Tx=Transplantation.

© 2023 Gaiffe E et al. *JAMA Network Open.*

eFigure 3. Correlation matrix between transfusion characteristics associated with transplant failure endpoint in univariate analyses

	RBC units	RBC episodes	Delay 1rst RBC unit	Min RBC storage duration	Min blood donor age	Mean delay for RBC transfusion
RBC units	1.00000	0.53319	-0.26078	-0.25187	-0.31119	-0.05765
	-	<0.0001	<0.0001	<0.0001	<0.0001	0.0007
RBC episodes	0.53319	1.00000	-0.29299	-0.22514	-0.26008	0.10385
	<0.0001	-	<0.0001	<0.0001	<0.0001	<0.0001
Delay 1rst RBC unit	-0.26078	-0.29299	1.00000	0.10131	0.13081	0.88799
	<0.0001	<0.0001	-	<0.0001	<0.0001	<0.0001
Min RBC storage duration	-0.25187	-0.22514	0.10131	1.00000	0.15738	0.00559
	<0.0001	<0.0001	<0.0001	-	<0.0001	0.7417
Min blood donor age	-0.31119	-0.26008	0.13081	0.15738	1.00000	0.01530
	<0.0001	<0.0001	<0.0001	<0.0001	-	0.3667
Mean delay for RBC transfusion	-0.05765	0.10385	0.88799	0.00559	0.01530	1.00000
	0.0007	<0.0001	<0.0001	0.7417	0.3667	-

Pearson correlation coefficients:	< 0.2	0.2-0.3	0.3-0.4	≥0.4
P-value:	> 0.001	≤ 0.001		

A significant correlation was considered for a correlation coefficient ≥ 0.4 associated with a P-value < 0.001: among “RBC units” and “RBC episodes”, the parameter “RBC episodes” is selected; among “Mean delay for RC transfusion” and “Delay 1rts RBC units”, the parameter “Delay 1rts RBC units” is selected. RBC=Red Blood Cell.

© 2023 Gaiffe E et al. *JAMA Network Open.*

eFigure 4. Kaplan-Meier transplant survival curves for patients according delay from Tx to issue of first red-cell unit transfusion or/and minimum duration of transfused red cells storage for transfused patients with one transfusion episode (A) or more (B).

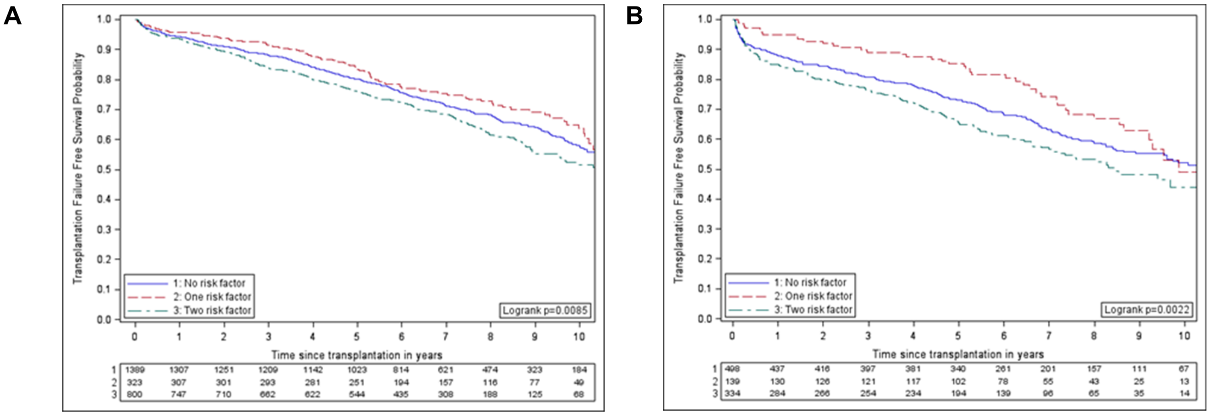

The transfusion parameters were analyzed in terms of risk factor: either the patient received a RBCT with a median for minimum duration storage greater than 20 days, or a RBCT after the transplant day, or both or neither. Tx=Transplantation, TXFFS=Transplantation Failure Free Survival.

© 2023 Gaiffe E et al. *JAMA Network Open.*

eFigure 5. Correlation matrix between transfusion characteristics associated with transplant failure endpoint in univariate for patients who have received one transfusion episode of one or two units

	Delay 1rst RBC unit	Min RBC storage duration	Min blood donor age	Mean delay for RBC transfusion
Delay 1rst RBC unit	1	0.00054	-0.04016	0.99983
	-	0.9810	0.0797	<0.0001
Min RBC storage duration	0.00054	1	0.04802	0.00009
	0.9810	-	0.0361	0.9969
Min blood donor age	-0.04016	0.04802	1	-0.03980
	0.0797	0.0361	-	0.0824
Mean delay for RBC transfusion	0.99983	0.00009	-0.03980	1
	<0.0001	0.9969	0.0824	-

Pearson correlation coefficients:	< 0.2	0.2-0.3	0.3-0.4	≥0.4
P-value:	> 0.001	≤ 0.001		

The significant correlations were defined by a correlation coefficient ≥ 0.4 associated with a P-value <0.001: among “Delay from transplantation to issue of first red-cell unit transfusion” and “Mean delay from transplantation to issue of red-cell unit for transfusion”, the parameter “Delay from transplantation to issue of first red-cell unit transfusion” is selected. RBC=Red Blood Cell.

© 2023 Gaiffe E et al. *JAMA Network Open.*
