# Supplementary material for: Transfused Red Blood Cell Characteristics and Kidney Transplant Outcomes Among Patients Receiving Early Posttransplant Transfusion
Source: JAMA Netw Open. 2023 Sep 14;6(9):e2332821. doi: 10.1001/jamanetworkopen.2023.32821 (PMC10502525; doi:10.1001/jamanetworkopen.2023.32821)
Supplement: Supplement 2. — Data Sharing Statement [file jamanetwopen-e2332821-s002.pdf]

Data Sharing Statement

Gaiffe. Transfused Red Blood Cell Characteristics and Kidney Transplant Outcomes Among Patients Receiving Early Posttransplant Transfusion. *JAMA Netw Open*. Published September 08, 2023. doi:10.1001/jamanetworkopen.2023.32821

Data
Data available: No

Additional Information
Explanation for why data not available: The raw data supporting the conclusions of this article will be made available by the authors, subject to the necessary regulatory procedures.
